# Individuals’ perceptions and experiences of mHealth for home-based rehabilitation in knee osteoarthritis: A qualitative study

**DOI:** 10.1016/j.ocarto.2026.100840

**Published:** 2026-06-15

**Authors:** Mathilde Pelletier Visa, Inès Monborne, Charlotte Lanhers, Sylvain Mathieu, Emmanuel Coudeyre

**Affiliations:** a Department of Physical Medicine and Rehabilitation, Clermont-Ferrand University Hospital, Louise Michel Hospital, Cébazat, France; b Human Nutrition Unit, INRAE, Clermont Auvergne University, France; c Neurodol, UMR 1017, Clermont Auvergne University, France; d Rheumatology Department, Clermont-Ferrand University Hospital, Clermont-Ferrand, France

**Keywords:** Knee osteoarthritis, Qualitative research, Patient acceptance, Rehabilitation, Telemedicine

## Abstract

**Objective:**

Individuals with knee osteoarthritis (KOA) rarely continue exercising after discharge from physiotherapy. We aimed to explore barriers and facilitators influencing the use of mHealth for home-based rehabilitation among people with KOA.

**Design:**

An inductive qualitative study using semi-structured interviews was conducted in a Physical and Rehabilitation Medicine outpatient department in France. Adults aged ≥40 years with physician-diagnosed KOA and prescribed rehabilitation were recruited. Interviews were performed face-to-face or by telephone, audio-recorded and transcribed verbatim. Data were analysed using an inductive thematic approach; two researchers independently coded transcripts and performed triangulation using NVivo software.

**Results:**

We included 26 participants (61.5% women; median [Q1; Q3] age 63.5 years [60.0; 73.5]). Median interview duration was 40 min 30 s [32min23; 48 min 15]. Five themes were identified: (1) Professional legitimisation as a prerequisite for mHealth adoption; (2) mHealth as a complement to physiotherapy; (3) Supported autonomy: balancing independence and human guidance; (4) Prior experience shapes perceptions but not expectations; (5) Usability, personalisation and perceived usefulness drive engagement. Participants generally had limited prior experience with mHealth. Engagement was influenced by low awareness, digital literacy challenges, and a need for supervision, but also by perceived potential benefits when tools were tailored and endorsed by healthcare professionally.

**Conclusions:**

Individuals with KOA appeared cautiously receptive to mHealth despite limited prior experience. Adoption depends less on technology itself than on professional endorsement, usability, and integration within existing care pathways. mHealth may support home-based rehabilitation when implemented as part of a supervised and structured care model.

## 1 Introduction

International guidelines from the European Alliance of Associations for Rheumatology [1] and the Osteoarthritis Research Society International [2] recommend non-pharmacological management of knee osteoarthritis (KOA). Supervised physiotherapy is a cornerstone of this management, including muscle strengthening, gait rehabilitation and mobility exercises [[3], [4], [5], [6]]. These interventions improve pain, knee flexion and extension, and overall physical function, enhancing performance of daily life activities [6]. However, once supervised physiotherapy ends, many individuals fail to continue performing regular exercises at home, leading to a progressive loss of the functional gains achieved [7]. Knee and hip OA represent a substantial socio-economic burden, with estimated mean annual total costs per individual of approximately 2.180€, and median population level costs of up to 2 billion € per year, driven by healthcare use and indirect costs (e.g., productivity losses) [8].

Mobile health (mHealth) technologies, including smartphone apps, have emerged as innovative tools to support exercise adherence in chronic musculoskeletal conditions [9,10]. MHealth are increasingly proposed to support home-based rehabilitation in KOA. In this study, we focused specifically on asynchronous mHealth interventions. Smartphone adoption rates now exceed 70% among older adults in Europe [11,12]; therefore, the use of apps among people with OA is feasible. These applications deliver tailored exercise programmes, monitor progress, and motivational support, potentially extending the rehabilitation benefits into the home [13]. However, little is known about how individuals with KOA perceive such tools, what drives or hinders their use, and how these digital resources can be integrated into care pathways.

Recent randomised controlled trials and meta-analyses have demonstrated that digital exercise programs can effectively reduce pain and improve function in people with KOA [14], with results comparable to supervised in-person programs and superior to unsupervised exercises [15,16]. Although the feasibility and clinical benefits have been reported, the successful implementation of these tools in routine clinical practice depends on complex human factors, including motivation, trust in digital content, usability, and professional support [17,18]. These psychosocial and relational dimensions are difficult to fully capture using quantitative approaches alone [19,20]. Similar findings were reported by Nelligan et al. [21], who showed that people with KOA valued eHealth interventions when they supported exercise adherence and were integrated with professional care. Despite the growing availability of mHealth for KOA, few tools have been rigorously validated, and there remains a significant evidence gap regarding how individuals' perceptions and lived experiences influence long-term adoption [22,23]. Addressing this gap is essential to optimise digital rehabilitation strategies and ensure their successful integration into clinical care. We aimed to explore patients’ perceptions, experiences, and expectations regarding the use of mHealth for home-based rehabilitation in KOA, with particular attention to factors influencing engagement, adoption, and long-term use.

## 2 Methods

### 2.1 Design

We conducted an inductive qualitative study using semi-structured interviews in the Physical Medicine and Rehabilitation (PMR) department at Clermont-Ferrand University Hospital between the 17th of June and the 29th of July 2025. The interview guide was iteratively adapted during data collection.

The study is reported according to the Consolidated Criteria for Reporting Qualitative Research guidelines [24].

### 2.2 Ethical considerations

This study was conducted outside the scope of the French “Research Involving Human Participants” regulations, as it did not involve any intervention or modification of the participants' usual care, according to the French Jardé Law of 2012. Ethical approval was obtained from the local Institutional Review Board of Clermont-Ferrand University Hospital (IRB00013412, *CHU de Clermont-Ferrand* IRB #1, No. 2025-CF466). All participants provided written informed consent in accordance with the French non-opposition procedure. Data collection and processing complied with the European General Data Protection Regulation; all transcripts were anonymised, and audio recordings were securely deleted after transcription.

### 2.3 Participants

Participants were recruited via a purposive, criterion based sampling strategy, prioritising informational richness over statistical representativeness from the PMR department at Clermont-Ferrand University Hospital. Information posters were used, and individuals treated in the unit were also contacted by phone. During these contact calls, the study's objectives and the researcher's background were clearly explained. This approach aimed to gather people with a range of experiences, including those familiar with mHealth apps, such as participants from the ARTH-e study [25] (see description below), and those unfamiliar with such apps. Before each semi-structured interview, participants received a detailed briefing note by email. The inclusion criteria were aged between 40 and 85 years; diagnosed with symptomatic KOA according to European Alliance of Associations for Rheumatology criteria [1]; with a prescription for rehabilitation; owning a smartphone/tablet; and having consented to participate in an interview.

Individuals with specific inflammatory pathologies (e.g., rheumatoid arthritis or spondyloarthritis), contraindications to physical activity, neurological conditions (e.g., stroke, multiple sclerosis or Parkinson's disease), difficulty understanding French, or who did not wish to participate were excluded.

#### 2.3.1 Arth-e study participants

Some individuals followed in our department had participated in the ARTH-e study (https://clinicaltrials.gov/study/NCT06359171). This study aimed to compare the effectiveness of the ARTH-e application combined with standard care versus standard care alone on exercise adherence, assessed using the EARS [25]. The ARTH-e application is a digital health solution, presently dedicated to individuals with KOA. This is an asynchronous mHealth intervention, providing self-directed exercise sessions without real-time supervision or direct interaction with healthcare professionals. It is currently used at our hospital for clinical research. Participants involved in clinical protocols download the tool onto their smartphone or tablet via the Google Play Store or the Apple App Store. To guarantee data security and confidentiality, the registration process requires scanning a QR code provided by our hospital, which enables anonymisation of the user profile before creating a secure account using an email address and password. The interface features an intuitive home screen including a dashboard, an educational “Did you know?” section to deepen knowledge of the pathology via quizzes (True/False), and direct access to the current exercise program. Each session lasts 25–30 min and is structured into five phases: a cardiovascular warm-up, a joint warm-up, muscle strengthening, neuromuscular control exercises (identified as “proprioception balance” within the interface), and stretching. To encourage engagement and adaptation to the individual's abilities, three levels of difficulty are offered as the user progresses.

### 2.4 Semi-structured interviews

We conducted 26 semi-structured interviews (10 by telephone and 16 face-to-face in a consultation room in the PMR department). All were led by a female PhD student (MPV), who holds a Master of Science degree and has experience in qualitative research. During the interviews, the researcher wore civilian clothing and was not identifiable as a clinician. The interviewer had received formal training in qualitative research, and the analysis was supervised by a senior researcher (EC) with experience in qualitative research Sixteen participants had prior exposure to the ARTH-e application through a previous research protocol [25], which may have influenced their perceptions and familiarity with mHealth tools. To address this potential bias, a comparative analysis was conducted between participants with and without prior ARTH-e exposure. This relationship was limited to research participation and did not involve therapeutic care. For the remaining participants, no prior relationship existed before study commencement. This semi-structured interview guide was initially developed by a physician (EC) and a Clinical Research Associate (MPV) (Appendix 1), and was subsequently revised and validated by another physician (CL) and another Clinical Research Associate (IM) to ensure clinical relevance and methodological rigour. The guide was pilot tested once before data collection to assess clarity, relevance, and comprehensibility. As no major modifications were required and the guide was focused and clinically grounded, one pilot interview was considered sufficient.

Interviews were audio-recorded using an iPhone 11, transferred immediately to a secure computer and transcribed verbatim on the same day. Audio files were then deleted from all devices. Transcripts were anonymised and assigned a unique identifier (P01–P26) with no link to personal identifiers. After the interviews, participants completed a socio-demographic questionnaire.

Recruitment and interviews continued until data saturation was achieved. Saturation was assessed progressively during data collection through regular review of transcripts and coding. It was considered reached when three consecutive interviews did not generate new code, themes, or significant analytical insights, and when existing categorieswere consistently repeated across individuals. Once the interviews were transcribed, the transcripts were emailed to the participants for any comments and/or corrections.

### 2.5 Data analysis

We performed an inductive thematic analysis using NVivo software (version 12; QSR International) to organise and systematise the data, without using a predefined theoretical framework. Initial codes were generated through repeated reading of verbatim transcripts using open coding, allowing concepts to emerge directly from participants’ narratives. These codes were then progressively grouped through axial and selective coding into broader categories and final themes. A secondary comparative analysis was conducted between participants previously exposed to the ARTH-e application and mHealth-naïve participants to explore whether prior experience influenced perceptions; however, this did not constitute a formal framework analysis. Two researchers (MPV, IM) independently coded all transcripts to ensure inter-coder reliability and minimise interpretation bias. Analytical triangulation was used to enhance credibility, and a reflexive logbook was maintained to ensure methodological transparency. Discrepancies were resolved through discussion and consensus; when needed, a third senior researcher (EC) was consulted to preserve analytical objectivity. Analytical triangulation, independent coding, a detailed reflexive logbook documenting methodological decisions, and potential researcher influences helped ensure transparency and rigour throughout the process. Rather than organising findings into pre-defined categories of barriers and facilitators, the analysis aimed to identify higher-order interpretative themes reflecting how participants understood and positioned mHealth within their rehabilitation pathways.

## 3 Results

### 3.1 Participants

**Fig. 1:**
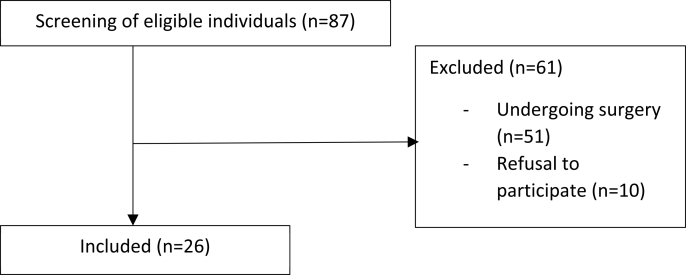
Flowchart of participant selection and inclusion.

Of 87 eligible individuals, 26 were included. Fig. 1 shows the selection process and exclusions. Interview duration ranged from 28 min 10 s to 55 min 40 s, with a median duration of 40 min 30 [32min23; 48 min 15]. Thematic recurrence was observed early in the data collection process, and no new concepts emerged in the final interviews, confirming that data saturation had been reached. The median [Q1; Q3] age of the 26 participants (10 men, 16 women) was 63.5 years [60.0; 73.5], and the median duration since the KOA diagnosis was 82 [63; 120] months. Most participants reported regular physical activity (89%), with more than half practicing at least four times per week. The median daily sedentary time was 4.75 h [3.25; 7.00], suggesting a relatively active sample. Participant characteristics are detailed in Table 1. Among them, 16 had previously used the ARTH-e application, and 6 reported prior use of other health applications (Table 2). Qualitative analysis of the interviews identified five main themes relating to participants’ perceptions of using mHealth within their rehabilitation. The five main themes and their corresponding subthemes are summarised in Table 3.

**Table 1:** Participant characteristics.

Characteristic	N = 26
Sex (female), n (%)	16 (61.5%)
Age (years), median [Q1; Q3]	63.5 [60.0; 73.5]
BMI (kg/m^2^), median [Q1; Q3]	26.99 [23.93; 30.45]
**Marital status,** n (%)	
Couple/single	14 (53.8%)/12 (46.2%)
Children	22 (84.6%)
**Level of education,** n (%)	
Less than high school certificate	5 (19%)
High school certificate	9 (35%)
Higher education	12 (46%)
**Living environment,** n (%)	
Urban	10 (39%)
Semi-rural	12 (46%)
Rural	4 (15%)
**Lifestyle,** n (%)	
Retired	18 (69%)
Active	8 (31%)
- Sedentary	1 (13%)
- Physical	3(38%)
- Mixed	4 (50%)
Daily sedentary time (hours), median [Q1; Q3]	4.75 [3.25; 7.00]
Regular physical activity**,** n (%)	23 (89%)
**Frequency**	
Every day	3 (13%)
4 to 6 times a week	9 (39%)
1 to 3 times a week	11 (48%)
Less than once a week	0 (0%)
Pain intensity over the past 24 h, median [Q1; Q3]	2 [0; 6]
**Affected knee,** n (%)	
Right	5 (19%)
Left	5 (19%)
Both	16 (62%)
Time since KOA diagnosis (months), median [Q1; Q3]	82 [63; 120]
**Area of OA lesions (from X-rays),** n (%)	
Medial tibiofemoral	21 (81%)
External tibiofemoral	6 (23%)
Patellofemoral	15 (58%)
**Radiological stage (Kellgrenn and Lawrence criteria),** n (%)	
Stage 1	0 (0%)
Stage 2	12 (46%)
Stage 3	7 (27%)
Stage 4	7 (27%)

**Table 2:** Characteristics of each participant.

	Age (years)	Sex	Lifestyle	Time since KOA diagnosis (months)	KOA stage	Participated in the ARTh-e Study	Previous use of an app
Participant 1	77	Female	Retired	53	Stage 4	Yes	No
Participant 2	63	Female	Retired	240	Stage 4	No	No
Participant 3	57	Male	Assets	84	Stage 2	No	Yes
Participant 4	60	Male	Retired	18	Stage 2	Yes	Yes
Participant 5	48	Male	Assets	72	Stage 2	Yes	No
Participant 6	74	Female	Retired	36	Stage 4	Yes	No
Participant 7	48	Female	Assets	72	Stage 3	Yes	Yes
Participant 8	77	Female	Retired	96	Stage 3	Yes	No
Participant 9	68	Male	Retired	120	Stage 3	No	No
Participant 10	75	Female	Retired	72	Stage 2	Yes	No
Participant 11	61	Female	Assets	120	Stage 4	No	No
Participant 12	60	Female	Assets	240	Stage 2	No	Yes
Participant 13	63	Male	Retired	204	Stage 4	No	No
Participant 14	68	Male	Assets	48	Stage2	No	No
Participant 15	70	Female	Retired	72	Stage 2	Yes	Yes
Participant 16	59	Female	Assets	84	Stage 4	Yes	No
Participant 17	76	Female	Retired	120	Stage 2	No	No
Participant 18	63	Female	Retired	60	Stage 2	Yes	No
Participant 19	77	Male	Retired	360	Stage 3	Yes	No
Participant 20	64	Male	Retired	48	Stage 4	Yes	Yes
Participant 21	76	Female	Retired	72	Stage 2	No	No
Participant 22	61	Male	Retired	360	Stage 2	No	No
Participant 23	72	Female	Retired	60	Stage 2	Yes	No
Participant 24	58	Female	Assets	80	Stage 3	Yes	Yes
Participant 25	71	Female	Retired	240	Stage 3	Yes	No
Participant 26	60	Male	Retired	84	Stage 3	Yes	Yes

**Table 3:** Main themes, subthemes, and illustrative quotes from the qualitative analysis.

Main theme	Subthemes
1. Professional legitimisation as a prerequisite for mHealth adoption	- Trust in healthcare professionals as a condition for app adoption - Lack of professional recommendation and low awareness of mHealth tools - Need for reassurance, supervision, and exercise validation - Concerns about safety and performing exercises alone
2. mHealth as a complement to physiotherapy, not a replacement	- Preference for face-to-face physiotherapy and human interaction - mHealth as a tool to maintain continuity of care at home - Use of apps between sessions or after discharge - Preference for a hybrid rehabilitation model combining physiotherapy and app-supported exercise
3. Supported autonomy: Balancing independence and human guidance	- Flexibility and autonomy in home-based rehabilitation - Self-management and personal responsibility in exercise practice - Fragility of motivation without external support - Need for reminders, feedback, and interpersonal encouragement
4. Prior experience shapes perceptions but not expectations	- Concrete feedback from ARTH-e users based on lived experience - Hypothetical expectations among participants without prior mHealth experience - Shared expectations regarding trust, personalisation, and professional endorsement - Prior exposure influencing appropriation rather than acceptance
5. Usability, personalisation, and perceived usefulness drive engagement	- Technical and ergonomic barriers (screen size, navigation, practicality) - Structured exercise sessions supporting routine formation - Perceived usefulness for pain reduction, mobility, and function - Monitoring, progress tracking, and motivation as drivers of sustained engagement

**Fig. 2:**
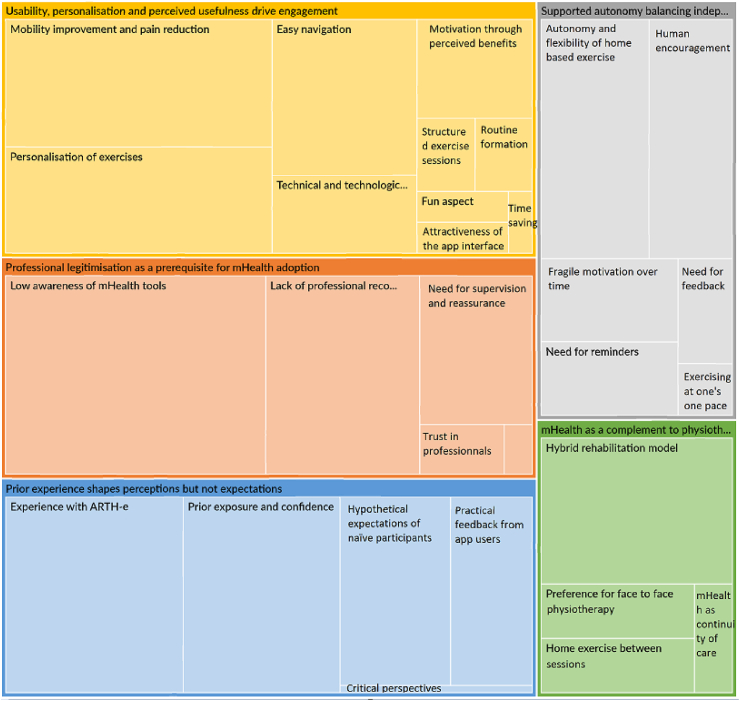
Thematic representation of barriers, levers and experiences reported by individuals. The size of the blocks is proportional to the recurrence of the themes identified (barriers to use, perceived benefits, relational dimensions and place within therapy). The empty boxes correspond to the fear of incorrect exercise performance in orange. **Yellow:** Usability, personalisation and perceived usefulness drive engagement; **Grey:** Supported autonomy: balancing independence and human guidance; **Orange:** Professional legitimisation as a prerequisite for mHealth adoption; **Green:** mHealth as a complement to physiotherapy, not a replacement; **Blue**: Prior experience shapes perceptions but not expectations.

Fig. 2 summarises the results of the qualitative analysis as a treemap generated using Nvivo software. The treemap illustrates the predominant themes reported by participants. The treemap provides a visual representation of the relative frequency of coded references; however, thematic importance was determined through interpretative analysis rather than frequency alone.

#### 3.1.1 THEME 1: professional legitimisation as a prerequisite for mHealth adoption

Healthcare professionals emerged as central actors in participants’ willingness to adopt mHealth for rehabilitation. Trust in clinicians strongly influenced perceptions of safety, legitimacy, and usefulness, with many participants considering professional recommendation as a prerequisite before engaging with a rehabilitation app. Most participants reported that neither their general practitioner, rheumatologist, nor physiotherapist had ever recommended a mobile application for KOA management. This absence of professional endorsement contributed to low awareness of available tools and reinforced scepticism regarding their clinical value.

*“Well, it's simple, I don't have any experience with it because I haven't been given any information about it.Let's be clear, neither by my general practitioner nor by my rheumatologist.” (P02)*

Participants also expressed concerns about performing exercises alone without supervision, particularly regarding movement accuracy, pain management, and the risk of worsening symptoms. Many emphasised the need for reassurance, feedback, and professional validation to feel confident using mHealth independently.

*“What’s holding me back is exactly that, not having someone right there to correct me if I’m doing the exercise wrong.*” (P01)

For most participants, mHealth was therefore perceived not as an autonomous solution, but as an extension of professional care requiring clinical guidance and legitimisation.

#### 3.1.2 THEME 2: mHealth as a complement to physiotherapy, not a replacement

Participants consistently positioned mHealth in relation to conventional physiotherapy rather than in opposition to it. While mobile applications were generally perceived as useful tools to support rehabilitation, they were rarely considered a substitute for face-to-face physiotherapy. Instead, participants viewed them as complementary resources that could extend therapeutic benefits between sessions or after discharge.

Many participants emphasised the importance of direct contact with healthcare professionals, particularly for correcting posture, adapting exercises to symptoms, and maintaining confidence throughout rehabilitation. In-person physiotherapy was associated with expertise, reassurance, and personalised follow-up that digital tools alone were not perceived to provide.

*“I prefer to do exercises with a physiotherapist, to have someone there to explain things to me and show me what to do.” (P01)*

MHealth was considered particularly valuable for maintaining continuity of care at home, especially when regular physiotherapy sessions were not possible because of time constraints, access difficulties, or the end of supervised rehabilitation. Participants described applications as a practical way to reinforce exercises already learned during physiotherapy and to maintain progress over time.

*“For me, it's not a barrier, it's a complement. In other words, I think physiotherapy is essential and the app could be a bonus, if it's well designed and well thought out.” (P14)*

Participants frequently described a combination of physiotherapy and app-supported home exercise as the preferred model, valuing mHealth as a transitional or supportive tool within an existing care pathway, rather than as a stand-alone intervention.

“*I think that could be something we could do more regularly, perhaps. And then we could have some targeted exercises that I could do at home when I'm not going to physiotherapy.” (P02)*

#### 3.1.3 THEME 3: supported autonomy: balancing independence and human guidance

Autonomy and flexibility were major facilitators of engagement with mHealth, particularly for participants seeking greater control over their rehabilitation. The possibility of exercising at home, at their own *pace*, and without the constraints of scheduled appointments was perceived as a significant advantage. This flexibility allowed rehabilitation to be more easily integrated into daily routines and adapted to individual preferences and physical condition.

*“You can do it however you want, whenever you want, whenever you feel like it, and you don't even have to leave the house. It's completely flexible. If you want to do it for half an hour, you do it for half an hour. If you want to do it for an hour, you do it for an hour. You can adapt it however you want, whenever you want, wherever you want. That's it.” (P15)*

Participants valued the freedom to repeat exercises as often as needed and to personalise their rehabilitation according to pain levels, fatigue, or daily availability. For some, this increased their sense of responsibility and active involvement in managing their condition. However, autonomy alone was not sufficient to sustain long-term engagement. Many participants described motivation as fragile and strongly dependent on external reinforcement, such as reminders, structured programmes, feedback, or follow-up from healthcare professionals. Without these support mechanisms, regular use often declined over time.

*“The problem is that I’m not motivated to do it on my own.” (P15)*

This tension revealed that participants did not seek complete independence, but rather supported autonomy, where self-management remained connected to professional guidance and interpersonal encouragement. Human contact was perceived as essential not only for technical reassurance, but also for maintaining commitment and confidence over time.

*“No, perhaps it's the feedback aspect that we don't get, which we can get with a physiotherapist.” (P24)*

However, the experience of autonomy appeared to be modulated by prior exposure to digital rehabilitation tools, which influenced how mHealth was perceived and appropriated.

#### 3.1.4 THEME 4: prior experience shapes perceptions but not expectations

Prior exposure to mHealth, particularly through the use of the ARTH-e application (n = 16), influenced the depth and concreteness of participants’ perceptions, rather than their overall acceptance of digital rehabilitation tools. A comparative analysis between participants with and without prior mHealth experience highlighted a shared recognition of the potential value of mobile applications for supporting rehabilitation, particularly in terms of motivation, exercise guidance, and self-management support (Table 4). However, differences emerged in the nature of discourse and experiential grounding. Participants with prior experience provided more concrete, practice-based evaluations, referring to usability, exercise variety, and integration into daily routines.

*“I've only tried one, ARTH-e, and I thought the app was well designed because it proposed a good variety of movements.” (P16)*

**Table 4:** Comparative thematic analysis between participants with prior ARTH-e use and mHealth-naïve participants.

Analytical theme	Participants with prior ARTh-e use (n = 16)	Participants without prior mHealth experience (n = 10)	Illustrative quotations
Professional legitimisation as a prerequisite for mHealth adoption	App accepted as an extension of professional care after introduction through the study; trust reinforced by prior supervised use	Strong expectation that mHealth should first be recommended and validated by healthcare professionals before adoption	**ARTH-e users**: “*You really do need guidance to perform the movements correctly, and it's true that the app makes perfect sense in situations like that.” (P04)***mHealth naïve**: “*Because a healthcare professional has expertise, qualifications, and experience. And an app, no matter how smart it may be, doesn't necessarily have those things.*” (P14)
mHealth as a complement to physiotherapy, not a replacement	App perceived as useful between sessions and after physiotherapy to maintain exercises and continuity of care	Strong preference for physiotherapy first; app considered acceptable only as a complement, never a replacement	**ARTH-e users:** “*It helps continue what we started with the physiotherapist.”* (P23) **mHealth naïve**: *“For me, it's not a barrier, it's a complement. In other words, I think physical therapy is essential, and the app could be a bonus, provided it's well-designed and well-thought-out.”* (P14)
Supported autonomy: Balancing independence and human guidance	Greater confidence in exercising independently once familiar with the app; autonomy seen as practical and motivating	Desire for autonomy but stronger need for reassurance, correction, and human feedback before engaging independently	ARTH-e users: *“You can do things at your own pace … as many times as you want.”* (P06) **mHealth naïve**: “*I also prefer to get professional feedback and have someone there in person to correct me and approve what I'm doing on the app*” (P12)
Prior experience shapes perceptions but not expectations	Concrete and practice-based evaluations of usability, exercise variety, and routine integration	More hypothetical expectations based on assumptions rather than lived experience	ARTH-e users: *“I thought the app was well designed because it proposed a good variety of movements.”* (P16) **mHealth naïve**: *“I wonder if that would actually work for me …”* (P22)
Usability, personalisation and perceived usefulness drive engagement	Benefits described through actual experience: pain reduction, mobility improvement, motivation, structured routine	Expected benefits focused on reassurance, progress monitoring, and guidance; stronger concerns about usability and exercise safety	ARTH-e users: *“It reduced pain and improved mobility*.” (P23) **mHealth naïve**: “*I hope to be more mobile.*” (P02)

In contrast, participants without prior mHealth experience expressed more hypothetical expectations. Their perceptions were mainly shaped by assumptions about what such tools could provide, particularly regarding motivation, exercise guidance, and progress monitoring. At the same time, they expressed stronger concerns about usability, technical complexity, exercise safety, and the absence of professional supervision.

Despite these differences, both groups shared similar expectations regarding trust, personalisation, and the need for healthcare professional endorsement. Prior exposure therefore influenced the depth and concreteness of perceptions rather than the overall acceptance of mHealth, suggesting that professional guidance remained central regardless of previous experience. This heterogeneity in prior experience further influenced how usability and therapeutic value were appraised in daily practice.

#### 3.1.5 THEME 5: usability, personalisation and perceived usefulness drive engagement

Participants’ engagement with mHealth solutions emerged from the interaction between usability, integration into daily routines, and perceived personal relevance of health benefits. Adoption was not driven solely by access to technology but by a process of appropriation shaped by ease of use and perceived clinical value.

However, technical and ergonomic constraints influenced sustained use. Some participants reported limitations linked to mobile device characteristics, particularly screen size, affecting practicality in home-based contexts.

*“Well, I did it when I had to, but it's true that I find on an app … I only have a smartphone, so it's a bit small … it's not … It's not the most practical. ∗crosses arms∗” (P0*1*)*

This suggests that acceptability depends not only on content relevance but also on the material conditions of use, which shape long-term adherence. Beyond technical aspects, clarity and structuring of exercises facilitated engagement by supporting routine formation. Conversely, irregular use was associated with declining motivation, reflecting fragile adherence in unsupervised contexts. Perceived usefulness was a key determinant of continued use. Participants engaged with the application not because it was available, but because it was perceived as meaningful for their condition.

*“Well, let's say that the app perhaps forces me to actually do it, rather than just taking the time to say to myself, Right, I need to do something. So there you go. And the benefit, in my opinion, is that the more exercise you do, the better.” (P01)*

This reflects externally supported self-regulation, where the application functions as a structuring tool for behaviour rather than a simple informational resource. Perceived benefits also strongly shaped engagement, particularly pain reduction, improved function, and sometimes reduced medication use. These anticipated outcomes acted as key motivational drivers.

“*To no longer have pain, to have well-developed leg muscles and, well, to no longer have any pain.”* (P26)

This illustrates how engagement is guided by direct projection of functional recovery goals onto rehabilitation expectations. In some cases, engagement was reinforced by a perception of monitoring and structured follow-up, with the application acting as a tool for progress tracking and motivation.

*“It's a check-up, it's knowing where we stand, it's motivation.” (P09)*

Overall, engagement with mHealth resulted from a continuous appraisal balancing usability, ease of integration, and perceived therapeutic value. When perceived as simple, accessible, and clinically meaningful, mHealth supported sustained adherence; when barriers or lack of immediate benefit were present, engagement declined.

Taken together, these themes describe a progressive and interdependent process of mHealth adoption, spanning from clinical legitimisation to sustained engagement in home-based rehabilitation.

## 4 Discussion

This study provides an individual-centred perspective on mHealth for KOA in a French rehabilitation context. Engagement with mHealth appeared to result from a progressive process involving clinical legitimisation, integration within rehabilitation pathways, supported autonomy, prior experience, and perceived usefulness, rather than technological features alone [14,26]. Healthcare professionals emerged as central actors in mHealth adoption [16]. Their endorsement strongly influenced participants’ trust, perceived safety, and willingness to engage, reinforcing digital tools as extensions of professional care rather than a standalone intervention [20,22]. These findings are consistent with Nelligan et al. [21], who also reported that patients viewed eHealth as a complement to physiotherapy rather than a replacement, and emphasised the importance of professional reassurance and sustained motivation. Despite recommendations supporting exercise and self-management in KOA [1,2,4], most participants reported never being advised to use mHealth, highlighting a persistent implementation gap [21,22].

Participants consistently preferred a hybrid rehabilitation model combining face-to-face physiotherapy with app-supported home exercise [21]. MHealth was perceived as useful for maintaining continuity of care between sessions or after discharge, but not sufficient to replace professional supervision. This is consistent with previous studies supporting blended care approaches in musculoskeletal rehabilitation, where digital tools enhance continuity of care without replacing therapeutic relationships [7,14,27,28].

Although autonomy and flexibility were valued, participants described a form of supported autonomy, where self-management remained dependent on reminders, feedback, and professional reassurance [27,29]. Motivation was often fragile without interpersonal support, confirming that adherence to home-based exercise remains challenging despite digital tools [9,10,15].

Prior exposure to the ARTH-e application influenced the concreteness of perceptions rather than overall acceptance. Experienced users provided more practical feedback, while app-naïve participants expressed more hypothetical expectations and concerns. However, both groups shared similar expectations regarding trust, personalisation, and clinician involvement, suggesting that prior experience shapes appropriation rather than acceptance [21,30].

Usability and perceived usefulness were major drivers of engagement. Technical barriers, ergonomic constraints, and lack of tailored feedback limited sustained use, whereas pain reduction, functional improvement, and progress monitoring reinforced adherence. These findings are consistent with previous studies on usability and digital literacy in older adults and KOA populations [12,14,31].

From a public health perspective, these findings are particularly relevant. Improving long-term adherence to exercise is a major challenge in KOA management and a key determinant of functional outcomes. Digital tools may help extend rehabilitation beyond supervised physiotherapy, support continuity of care, and potentially reduce healthcare utilisation associated with symptom progression and functional decline [32,33].

Overall, mHealth was perceived as a complementary and transitional tool embedded within a broader rehabilitation pathway. A structured hybrid model including initial face-to-face assessment, guided app onboarding, and follow-up may better support long-term adherence while preserving safety and therapeutic alliance [27,28].

Several limitations should be acknowledged. First, the monocentric design conducted in a tertiary care rehabilitation department in France may limit transferability to other healthcare settings, particularly primary care. Second, more than half of the participants reported engaging in physical activity at least four times per week. This relatively active profile may not reflect the broader population of people with KOA, which is often less physically active, and may have led to an overestimation of motivation toward mHealth-based rehabilitation and acceptance of self-managed exercise. Future studies should include less active patients to further explore barriers to adoption. Third, prior exposure to the ARTH-e application among 16 participants may have influenced perceptions. However, this was addressed through comparative analysis, independent coding, analytical triangulation, and reflexive documentation, and prior exposure appeared to influence the depth of perceptions rather than their overall direction.

## 5 Conclusions

This qualitative study suggests that, although individuals with KOA are generally unfamiliar with mHealth for rehabilitation, they perceive clear benefits when these tools are used, particularly for pain management, mobility, motivation, and self-management. Engagement with mHealth depends less on technology itself than on professional legitimisation, usability, and integration within existing rehabilitation pathways. MHealth appears to represent a complementary approach to conventional rehabilitation rather than a replacement, particularly when tools are adapted, personalised, and supported by healthcare professionals. The limited recommendation of these tools by professionnals remains a major barrier to adoption, highlighting a persistent gap between digital innovation and routine clinical practice. Our findings suggest that hybrid care models combining professional supervision with app-supported home rehabilitation may better align with patient expectations than fully autonomous use.

## Authors contributions

MPV designed the study and developed the semi-structured interview guide with EC. CL and IM contributed to the critical revision of the interview guide. MPV conducted the interviews, collected and analysed the data. MPV and IM wrote the first draft of the manuscript. All authors contributed to the interpretation of the results, revised the manuscript critically for important intellectual content, and approved the final version.

## AI use statement

In this manuscript, artificial intelligence was used to rephrase and streamline certain sentences.

## Funding source

None.

## Conflict of interest

The authors declare that they have no conflicts of interest.
