# Supplementary material for: Individuals’ perceptions and experiences of mHealth for home-based rehabilitation in knee osteoarthritis: A qualitative study
Source: Osteoarthr Cartil Open. 2026 Jun 15;8(3):100840. doi: 10.1016/j.ocarto.2026.100840 (PMC13314908; doi:10.1016/j.ocarto.2026.100840)
Supplement: Multimedia component 1 [file mmc1.docx]

**Appendix 1.**  Semi-structured interview grid

1. Can you tell me about your experience using digital applications or programs for your health?
2. Do you currently have any health-related apps installed on your phone or tablet? If so, which ones and how do you use them?
3. Can you give me a list of specific applications for joint or knee rehabilitation?
4. Can you tell me what you know about home rehabilitation applications?
5. How often do you use a rehabilitation application, and in what circumstances?
6. How do you think an app could help you keep better track of your rehabilitation? What would be the benefits?
7. What difficulties have you encountered in finding or using a suitable rehabilitation application?
8. Has a healthcare professional ever advised you to use an application for your rehabilitation? If so, what happened?
9. What attracts or inhibits you from using a mobile application for your rehabilitation, as opposed to supervised face-to-face exercises?
10. If a medical specialist recommended a rehabilitation application, how would you feel about trying it out? What would encourage or discourage you?
